# Oxidation precursor dependence of atomic layer deposited Al_2_O_3_ films in a-Si:H(i)/Al_2_O_3_ surface passivation stacks

**DOI:** 10.1186/s11671-015-0798-2

**Published:** 2015-03-19

**Authors:** Yuren Xiang, Chunlan Zhou, Endong Jia, Wenjing Wang

**Affiliations:** Key Laboratory of Solar Thermal Energy and Photovoltaic System, Institute of Electrical Engineering, Chinese Academy of Sciences, No. 6 Beiertiao, Zhongguancun, Beijing, 100190 China

**Keywords:** Atomic layer deposition, Al_2_O_3_, A-Si:H(i)/Al_2_O_3_ stack, Interface trap density

## Abstract

In order to obtain a good passivation of a silicon surface, more and more stack passivation schemes have been used in high-efficiency silicon solar cell fabrication. In this work, we prepared a-Si:H(i)/Al_2_O_3_ stacks on KOH solution-polished n-type solar grade mono-silicon(100) wafers. For the Al_2_O_3_ film deposition, both thermal atomic layer deposition (T-ALD) and plasma enhanced atomic layer deposition (PE-ALD) were used. Interface trap density spectra were obtained for Si passivation with a-Si films and a-Si:H(i)/Al_2_O_3_ stacks by a non-contact corona C-V technique. After the fabrication of a-Si:H(i)/Al_2_O_3_ stacks, the minimum interface trap density was reduced from original 3 × 10^12^ to 1 × 10^12^ cm^−2^ eV^−1^, the surface total charge density increased by nearly one order of magnitude for PE-ALD samples and about 0.4 × 10^12^ cm^−2^ for a T-ALD sample, and the carrier lifetimes increased by a factor of three (from about 10 μs to about 30 μs). Combining these results with an X-ray photoelectron spectroscopy analysis, we discussed the influence of an oxidation precursor for ALD Al_2_O_3_ deposition on Al_2_O_3_ single layers and a-Si:H(i)/Al_2_O_3_ stack surface passivation from field-effect passivation and chemical passivation perspectives. In addition, the influence of the stack fabrication process on the a-Si film structure was also discussed in this study.

## Background

An excellent interface passivation has been considered as the key point for high-efficiency solar cells such as passivated emitter and rear cell (PERC), heterojunction with intrinsic thin layer (HIT) or interdigitated back contact (IBC) device structures. The properties of aluminum oxide (Al_2_O_3_) films and hydrogenated amorphous silicon (a-Si:H) films have been widely investigated for solar cell fabrication. Both of them have shown excellent performances, such as remarkable passivation behavior on both n- and p-type Si surfaces and the cost-saving deposition using atomic layer deposition (ALD) and plasma-enhanced vapor chemical deposition (PECVD) at low temperatures, respectively [1]. The effective passivation of Al_2_O_3_ film is related to the field-effect passivation associated with the fixed negative charges (*Q*_f_, about 3–10 × 10^12^ cm^−2^) and chemical passivation associated with low interface trap density (*D*_it_, ≤10^11^ eV^−1.^cm^−2^) [2]. The passivation effect of a-Si:H films with low *D*_it_ is generally attributed to the saturation of dangling bonds on Si surface by hydrogen [3].

Various passivation stack schemes, such as SiO_2_/Al_2_O_3_, Al_2_O_3_/a-SiN*x*:H, a-Si:H/SiN*x*, etc., have already been investigated to improve the passivation effect, giving consideration to both low temperature deposition process and stability of thermal and ultraviolet (UV) radiation in the photovoltaic field [4-7]. In SiO_2_/Al_2_O_3_ stacks, Al_2_O_3_ films play the role of a capping layer to improve the passivation effect [4]. The low *D*_it_ values obtained through this passivation scheme were explained by an effective hydrogenation of defects present at the buried Si/SiO_2_ interface under the influence of the Al_2_O_3_ capping layer [4,8]. In Al_2_O_3_/a-SiN*x*:H and Al_2_O_3_/SiN*x* stacks, Al_2_O_3_ films have been applied as an interface layer due to the excellent surface passivation and parasitic shunting free on p-type silicon, e.g., PERC [5,9-11]. A-Si:H/SiO*x* [12], a-Si:H/SiN*x* [13], and a-Si:H/SiN*x*:H [7] stacks have been studied as stable alternatives to thermally grown SiO_2_ because of the low a-Si:H deposition temperature. Those schemes use a-Si:H films as interface layers to ensure the relatively high quality passivation and eliminate the parasitic shunting in case of directly deposited SiN*x* or SiN*x*:H films on p-type silicon surface with parasitic shunting [7,14].

In this paper, a-Si:H(i)/Al_2_O_3_ passivation stacks for silicon were chosen as a model system for studying the passivation effect of the ALD Al_2_O_3_ films. It is surmised that the a-Si:H(i) films act as a hydrogen reservoir to improve the interfacial chemical passivation during the a-Si:H(i)/Al_2_O_3_ stack annealing. For the stack preparation, a-Si:H(i) films were deposited on just one side of silicon wafer by PECVD; after annealing of the wafers, ALD Al_2_O_3_ films were deposited on top of the a-Si:H layers. For reference purposes, the passivation of Al_2_O_3_ single layers on high-quality n-type Si wafers (without an intermediate a-Si:H layer) was also shown in this study. Combining the passivation testing results with an X-ray photoelectron spectroscopy (XPS) analysis, we discussed the influence of oxidation precursor for ALD Al_2_O_3_ deposition on Al_2_O_3_ single layer and a-Si:H(i)/Al_2_O_3_ stack surface passivation from field passivation and chemical passivation perspectives.

## Methods

**Figure 1:**
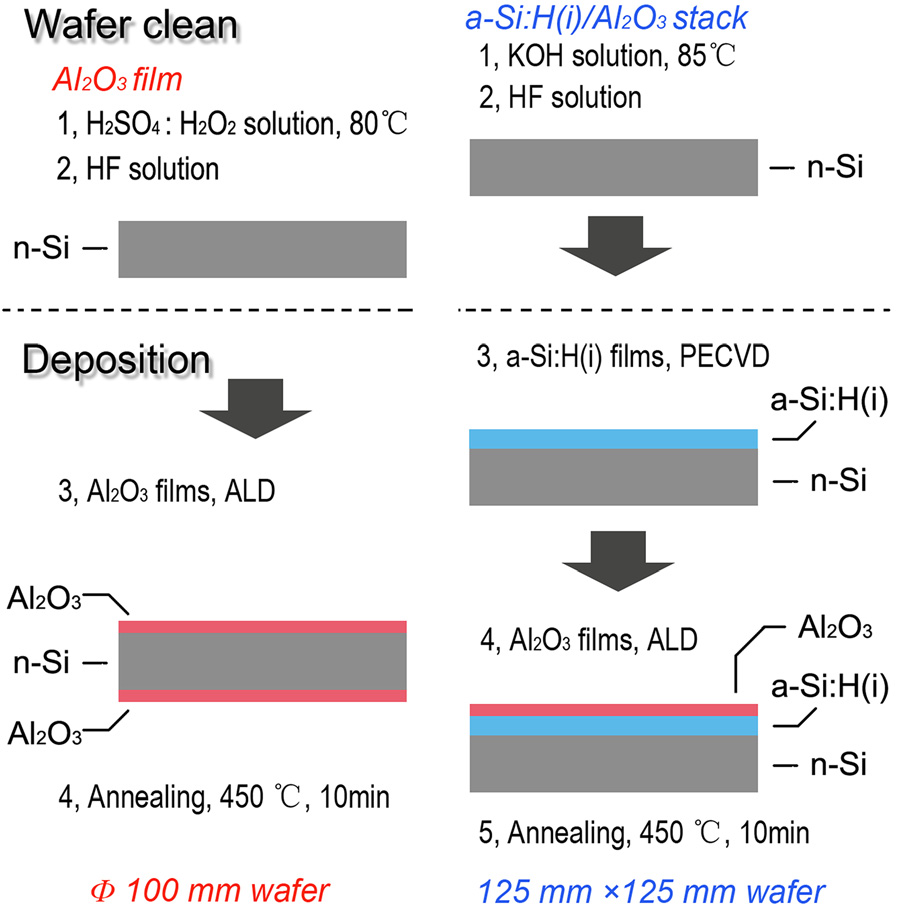
**Schematic representation of sample preparation.**

For the a-Si/Al_2_O_3_ stack passivation samples, 125 mm × 125 mm 0.9-Ω · cm phosphorus-doped solar grade mono-crystalline silicon(100) wafers (Jinglong Industry and Commerce Group Co. Ltd., Xingtai, Hebei Province, China) were used as substrates. The wafers were polished in KOH solution (Arkonic Gases & Chemicals Inc., Wuhu, Anhui Province, China) to a thickness of 160 μm and cleansed by a hydrogen fluoride (HF) solution (Arkonic Gases & Chemicals Inc., Wuhu, Anhui Province, China) to remove the native oxide layer. a-Si:H(i) films with thicknesses of 80 and 170 nm were deposited on just one side of the Si wafers by PECVD at 160°C using hydrogen (H_2_) and silane (SiH_4_) as precursor gases. Those films were annealed at 250°C for 10 min in air. For the reference Al_2_O_3_ single layer passivation samples, Ф100-mm polished n-type high quality silicon wafers were used as substrates. All the wafers were cleaned by H_2_SO_4_:H_2_O_2_ solution (4:1 vol) at 80°C. Before the Al_2_O_3_ film deposition, the native oxide layer present on the wafer surfaces was also removed using an HF solution. For the Al_2_O_3_ film deposition, both plasma-enhanced atomic layer deposition (PE-ALD) and thermal atomic layer deposition (T-ALD) were used. The ALD substrate temperatures were 200°C for the a-Si/Al_2_O_3_ stacks, while 100°C and 200°C for Al_2_O_3_ single layers. Al(CH_3_)_3_ (trimethylaluminum, TMA; Jiangsu Nata Opto-electronic Material Co., Ltd., Suzhou, Jiangsu Province, China) served as Al precursor, and either remote O plasma (for PE-ALD) or H_2_O (for T-ALD) was used as O precursor. After Al_2_O_3_ layer deposition, all the samples (both the Al_2_O_3_ single layers and the a-Si/Al_2_O_3_ stacks) were annealed at 450°C for 10 min in air. In addition, only the Al_2_O_3_ single layer passivation samples were covered with Al_2_O_3_ films (Jiangsu Nata Opto-electronic Material Co., Ltd., Suzhou, Jiangsu Province, China) on both sides of Si wafers. The sample preparation process was shown in Figure 1. The thickness of films was measured by a step profiler. The composition of Al_2_O_3_ films was measured by XPS (Beijing Synchrotron Radiation Facility, Beijing, China) before and after annealing. The interface trap density spectra were obtained from a non-contact corona C-V technique for a-Si single films and a-Si:H(i)/Al_2_O_3_ stacks (Institute of Electrical Engineering, CAS, Beijing, China) coating Si surface.

In this study, the X-ray photoelectron spectroscopy used 700-eV synchrotron-based light as X-ray radiation source and the adventitious C 1 s signal (at 284.6 eV) to calibrate the peak positions. Al 2p and O 1 s core-level spectra were measured in order to investigate the chemical state of the Al_2_O_3_ films.

The non-contact corona C-V technology is based on charge biasing of the dielectric with a precise charge dose, Δ*Q*_C_, deposited by a corona discharge in air. The corresponding surface voltage change including the change in surface barrier, *V*_SB_, is measured with a vibrating Kelvin probe. This technology monitors the interface trap density (*D*_it_) spectra, *D*_it_ vs. *V*_SB_ position in the Si energy gap, and the total charge density by means of the multi-metrology Semilab PV-2000 platform (Semilab Co. Ltd., Shanghai, China) [15]. The effective minority carrier lifetime was measured by the Semilab WT2000 set-up (Semilab Co. Ltd., Shanghai, China).

## Results and discussion

### The difference in the passivation between PE-ALD and T-ALD deposition

**Figure 2:**
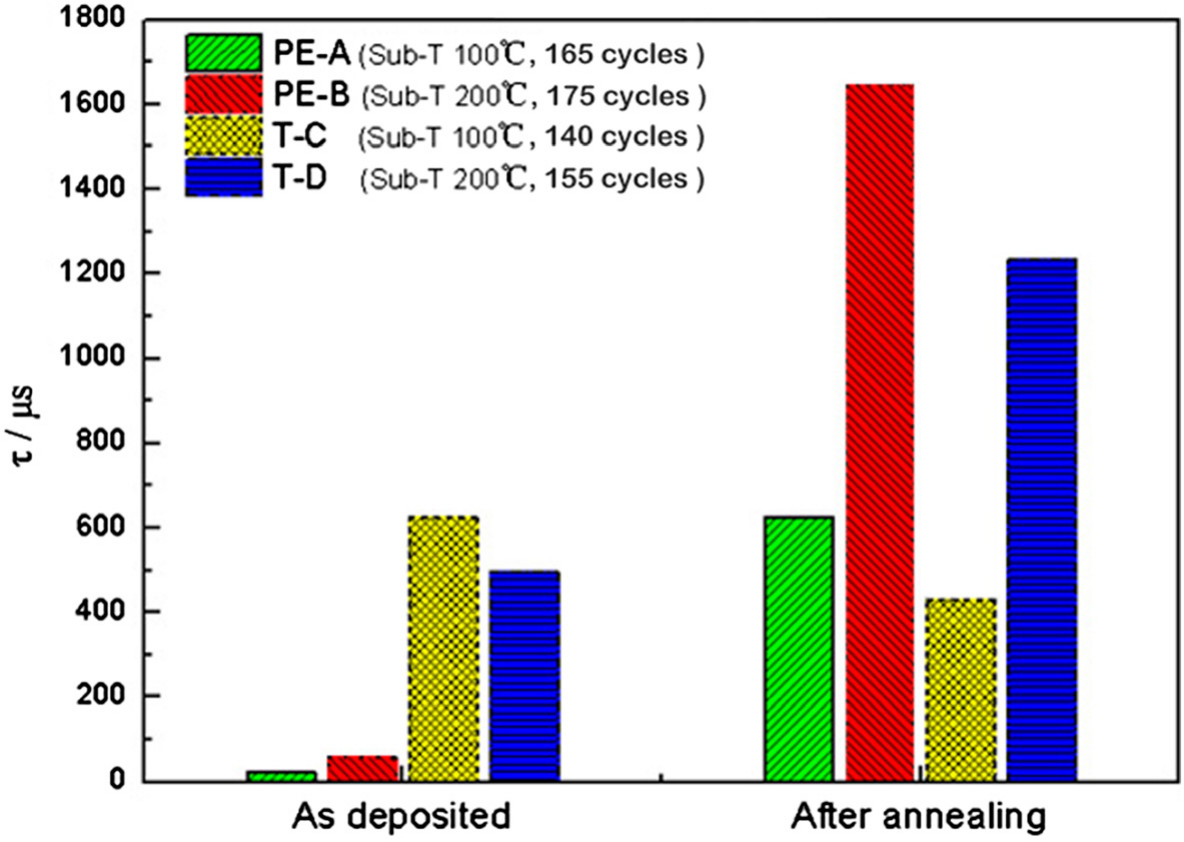
**Effective lifetime for n-type Si capped with Al**_**2**_ **O**_**3**_ **films before and after annealing.** The thickness of Al_2_O_3_ films is about 20 nm and the annealing process is done at 450°C for 10 min in air. The substrate temperature (Sub-*T*) and the number of ALD cycles are also shown here.

For the reference samples (Ф100 mm) covered by Al_2_O_3_ films on both sides, the effective minority carrier lifetimes before and after annealing are shown in Figure 2. The T-ALD samples have higher effective minority carrier lifetimes just before deposition, but the advantage is lost after annealing contrasted with PE-ALD samples, especially for the Al_2_O_3_ deposition at the low substrate temperature of 100°C. The samples with the high substrate temperature of 200°C for both deposition types show higher effective minority carrier lifetimes after annealing (1,647 μs for PE-ALD deposition, 1,232 μs for T-ALD deposition). The reasons for the different performances in the effective minority carrier lifetime for the two ALD deposition Al_2_O_3_ films are considered as follows: First, *D*_it_ of PE-ALD Al_2_O_3_ film-passivated Si surfaces is higher than that of T-ALD Al_2_O_3_ film-passivated Si surfaces due to the presence of vacuum ultraviolet radiation in the O_2_ plasma. Thus PE-ALD interfaces need an annealing step to improve performance. Second, the fixed charge densities in T-ALD Al_2_O_3_ films are lower than those in PE-ALD Al_2_O_3_ films [2]. The fixed charge density is related to field-effect passivation. The origin of the negative fixed charge in the Al_2_O_3_ film is considered to be related to the effect of the interfacial SiO*x* film which is formed during the ALD deposition [16].

Moreover, the T-ALD Al_2_O_3_ films with a substrate temperature of 100°C have a passivation degradation after annealing (see Figure 2). Due to the reaction between H_2_O and TMA, the Al_2_O_3_ film quality depends on the reaction activity of H_2_O and changes according to the variation of substrate temperature [17]. A lower substrate deposition temperature leads to a lower Al_2_O_3_ film quality. This degradation may be caused by loosely bound H atoms escaping from the loose interfacial layer by thermal driving.

### Chemical composition analysis of Al_2_O_3_ layer using XPS

**Figure 3:**
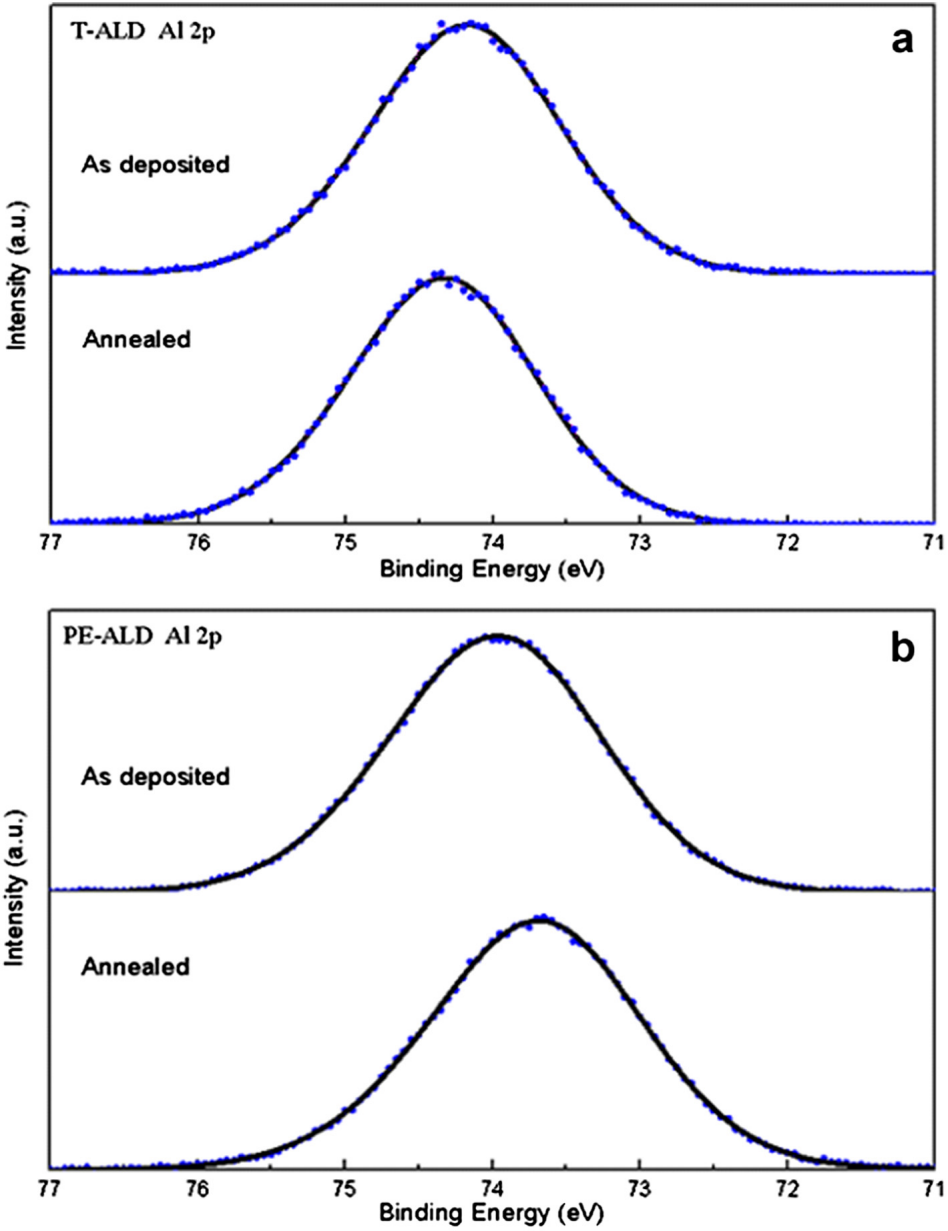
**Al 2p XPS spectra of Al**_**2**_ **O**_**3**_ **films.** The films were deposited by T-ALD **(a)** and by PE-ALD **(b)**. The solid black lines represent the fitted curves.

The difference in the chemical binding state between PE-ALD and T-ALD deposition (substrate temperature of 200°C) and the effect of annealing process on the chemical structure of the Al_2_O_3_ films were investigated by XPS measurement to study the passivation mechanism. Figure 3a,b shows the Al 2p core level spectra of Al_2_O_3_ films deposited by PE-ALD and T-ALD before and after the annealing process, respectively. Peaks de-convolution are not processed for analysis in here because the Al 2p position of AlOOH or Al(OH)_3_ is similar with that of Al_2_O_3_ [18]. Al 2p peak positions of as-deposited films are almost 74.1 eV irrespective of ALD type. After annealing at 450°C for 10 min in air, the positions shift to 74.3 eV (T-ALD) and 73.7 eV (PE-ALD). Compared with the reported Al 2p peak position of ALD Al_2_O_3_ films [19,20], those position shifts may be attributed to the charge accumulation, especially for the T-ALD high energy direction shift.

**Figure 4:**
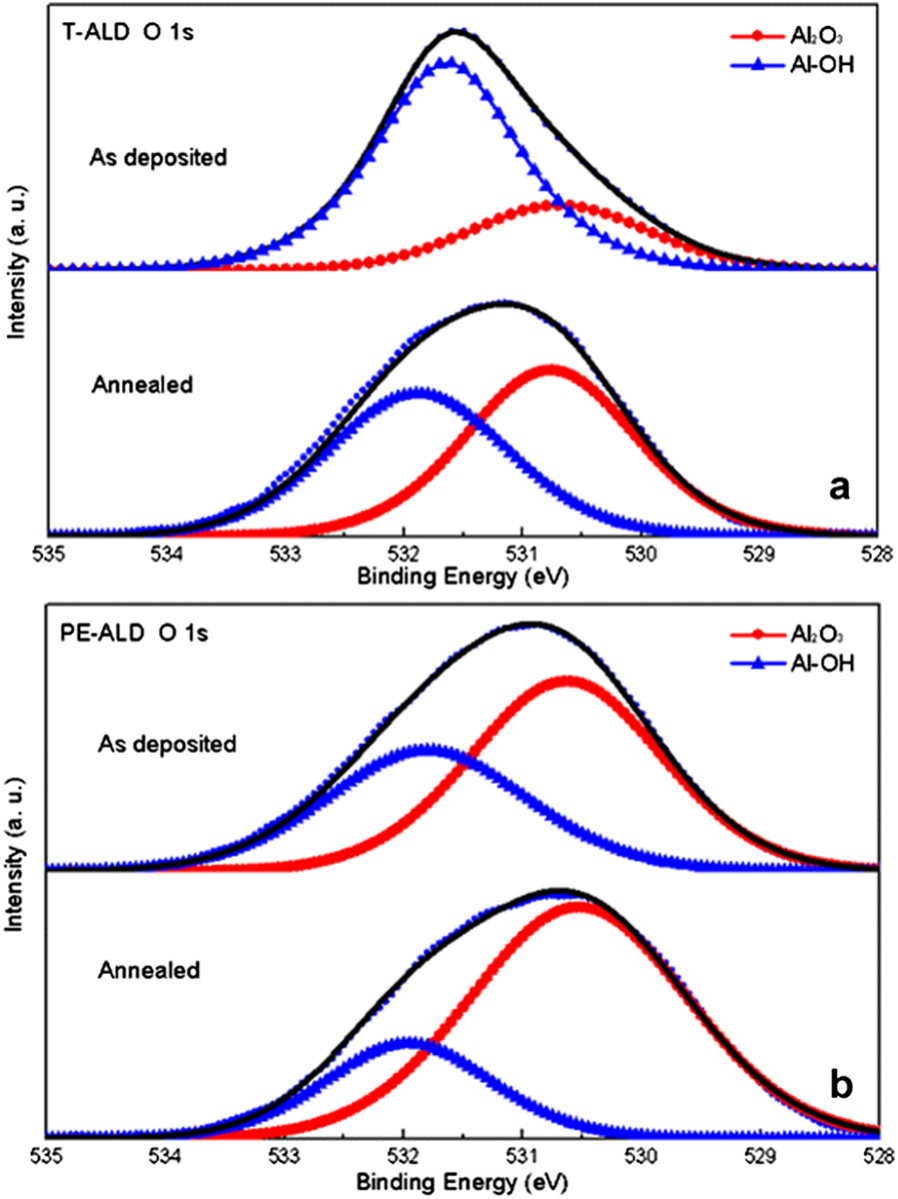
**O 1 s XPS spectra of Al**_**2**_ **O**_**3**_ **films.** Films were deposited by T-ALD **(a)** and by PE-ALD **(b)**. The solid black lines represent the fitted curves.

Figure 4a,b show the O 1 s core level spectra of Al_2_O_3_ films deposited by PE-ALD and T-ALD before and after annealing. Two components are de-convoluted form O 1 s peak - one centered at approximately 531.8 eV corresponding to Al-OH of AlOOH or Al(OH)_3_ and the other centered at approximately 530.7 eV corresponding to O-Al-O bonds of Al_2_O_3_. The fitted component curves (Al_2_O_3_, red line) in Figure 4b show that O-Al-O bonds have a higher occupation area than Al-OH bonds (Al-OH, blue line) in the PE-ALD-deposited film, and the area ratio (*A*_Al2O3_/*A*_Al-OH_) increases from 3:2 to 3:1 after annealing. By contrast with PE-ALD film, the area ratio of the T-ALD-deposited film is lower. But the variation trends of PE-ALD- and T-ALD-deposited films are similar after annealing, and the area ratio of the T-ALD film also increases from 3:7 to 5:4, showed in Figure 4a. The increase of the T-ALD area ratio after annealing is attributed to the residual O-H bonds breaking at high temperature. The bond breaking releases the interstitial H atoms which diffuse into the Al_2_O_3_ film and the Al_2_O_3_/Si interface [21], and saturate the dangling bonds of the silicon surface as discussed above. Simultaneously, the residual O-H bond breaking also releases Al atoms to form more O-Al-O bonds of Al_2_O_3_. By contrast with the PE-ALD film, the lower area ratio of the T-ALD film indicates that the reaction activity of H_2_O is lower than that of plasma O and residual OH group absorbed during reaction between TMA and H_2_O. In another aspect, the relatively stable area ratios for PE-ALD film before and after annealing are due to the high reaction activity of plasma O, which allows for a higher reaction ratio with TMA.

### The passivation effect of a-Si:H(i)/Al_2_O_3_ stack on mc-Si(n)

**Figure 5:**
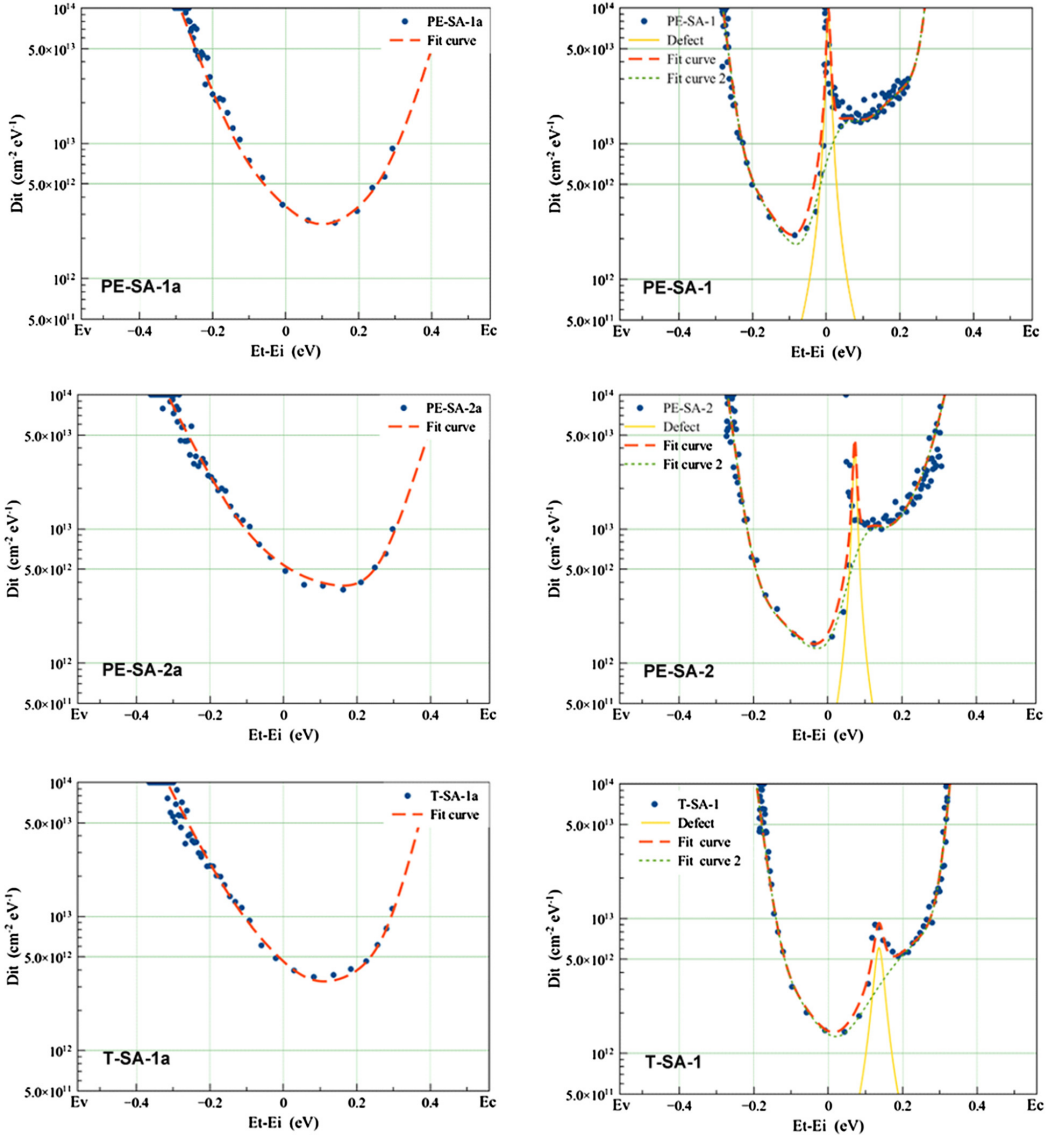
***D*** **it spectra of a-Si:H (left side) and Al**_**2**_ **O**_**3**_ **/a-Si:H stack (right side).** The Al_2_O_3_ films deposited on PE-SA-1 and PE-SA-2 by PE-ALD at 200°C. The Al_2_O_3_ films deposited on T-SA-1 by T-ALD at 200°C. PE-SA-1a, PE-SA-2a, and T-SA-1a are the samples only covered by the a-Si:H films and PE-SA-1, PE-SA-2, and T-SA-1 refer to the stack structure samples. The a-Si:H(i)/Al_2_O_3_ stack samples (the structures of samples are shown in Table 1) have been annealed at 450°C for 10 min in air. ‘Dots’ are the measured data by the non-contact corona C-V technology. ‘Fit curve’ are fitted curves for the measured data using the simplified density of an interface state model, which can been de-convoluted to ‘Defect’ and ‘Fit curve 2’.

Figure 5 shows the *D*_it_ spectra data of a-Si:H/Si interface with and without Al_2_O_3_ layer (the structures of samples are showed in Table 1). The directly measured voltage scale has been changed into energy scale referred to the silicon band edges. This conversion from the surface barrier (*V*_SB_) to silicon bulk energy level is based on [22] the following: i.) the Fermi level position of the n-type silicon wafer using in this study is (*Ef* − *Ei*) = 0.34 eV, where *Ei* refers to midgap (*E*_g_ = 1.12 eV), and ii.) the corresponding energy scale for the *D*_it_ spectra is (*Et* − *Ei*) = ((*Ef* − *Ei*) − *V*_SB_). So, it is obvious that (*Et* − *Ei*) = (*Ef* − *Ei*) = 0.34 eV in the flat-band condition (*V*_SB_ = 0). A simplified interface state model has been used to fit the directly measured data (dots in the Figure 5). This model consists of the valence and conduction band tails originating from weak Si-Si bonds and a dangling-bond distribution with two Gaussian components, which correspond to donor and accepter state in the a-Si:H(i)/Si interface [23]. ‘Fit curve’ shown in Figure 5 are fitted results using the simplified interface state density model, which can been de-convoluted to the discrete energy trap (‘Defect’) and ‘Fit curve 2’.

**Table 1:** **The structures of samples**

	**Before deposition**	**After deposition**
PE-SA-1	80 nm a-Si:H/Si	30 nm Al_2_O_3_ (210 cycles) + 80 nm a-Si:H/Si
PE-SA-2	80 nm a-Si:H/Si	10 nm Al_2_O_3_ (85 cycles) + 80 nm a-Si:H/Si
T-SA-1	170 nm a-Si:H/Si	30 nm Al_2_O_3_ (200 cycles) + 170 nm a-Si:H/Si

The numbers of ALD deposition cycles are shown behind the Al_2_O_3_ film thickness.

From the *D*_it_ spectra of a-Si:H(i)/Si interface, the three different samples have the same interface state density distribution and all get their lowest point (*D*_it_ < 4.0 × 10^12^ cm^−2^ · eV^−1^) at about 0.1 eV above midgap. The fitted curves show that the spectra have a trend to be asymmetric, and curves change slower with energy level in the side near to valence band than the side near conduction band. After the Al_2_O_3_ film deposition and annealing, the curves change faster in both sides near the conduction and valence bands and have lower minimum interface trap density (Min-*D*_it_) than the samples without Al_2_O_3_ film deposition. Those may be attributed to two reasons: first, the thermal process releases the stress of weak Si-Si bonds to decrease the density of the band tail stats; second, the H diffusion shifts the weak Si-Si bonds to dangling-bonds with H saturation.

It is worth noting that the *D*_it_ spectra of samples deposited with Al_2_O_3_ film seem to have two different regions, one with a low *D*_it_ bottom (Min-*D*_it_ ≤ 1 × 10^12^ cm^−2^ · eV^−1^) and anther with a high *D*_it_ bottom (Min-*D*_it_ ≥ 1 × 10^12^ cm^−2^ · eV^−1^). An asymmetric curve of the bulk density of states calculated by Winer [24] and Powell [25] is similar with the ‘Fit curve 2’ in Figure 5, and in their model, this curve is referred to as doped amorphous silicon (p type).

The discrete energy trap (‘Defect’ in Figure 5) has an energy level position at *E*_*t*_ − *E*_*i*_ = 0.14 eV for T-SA-1, at the middle of the energy gap *E*_*t*_ − *E*_*i*_ = 0 eV for PE-SA-1, and at *E*_*t*_ − *E*_*i*_ = 0.07 eV for PE-SA-2. The largest peak density of the trap in PE-ALD samples is higher than the that in T-ALD sample, and the PE-ALD defect peak shapes are sharp. It is well known that the defects in the middle of the Si energy gap can be called deep level centers, which will lead to an effective minority carrier lifetime reduction.

The field-effect passivation effect as a function of the surface total charges (*Q*_tot_, including the charged on the film top surface, in the film and interface) was detected by the Q-V measurement (see Table 2). In this study, *Q*_tot_ of each sample are negative. After Al_2_O_3_ film deposited, *Q*_tot_ increased by near one order of magnitude for PE-ALD samples and about 0.4 × 10^12^ cm^−2^ for T-ALD sample. The negative charges of a-Si:H(i)/Si originate from the electrons occupying the band gap states of donor and accepter energy level. Those electrons diffuse from n-type silicon wafer into a-Si:H(i) films caused by the energy level mismatch [26]. The negative charge density increase for the a-Si:H(i)/Al_2_O_3_ stacks may be mostly benefit from the large amount of negative charges in Al_2_O_3_ films. The PE-ALD Al_2_O_3_ films show more charges than T-ALD films in Table 2. PE-ALD precursor plasma O reaction with TMA shows a better property for negative charges supply, which agrees with the Al_2_O_3_ single layer passivation data and the XPS analysis above.

**Table 2:** **Electrical interface parameters**

		**PE-SA-1**	**PE-SA-2**	**T-SA-1**
Before deposition^a^	*Q*_tot_ (cm^−2^)	−9.23 × 10^11^	−7.70 × 10^11^	−9.93 × 10^11^
	*V*_SB_ (V)	−0.177	−0.134	−0.169
After deposition	*Q*_tot_ (cm^−2^)	−8.93 × 10^12^	−5.64 × 10^12^	−1.38 × 10^12^
	*V*_SB_ (V)	−0.546	−0.499	−0.192

^a^The samples before deposition refer to the samples only covered by a-Si:H films. The samples after deposition were annealed at 450°C for 10 min in air.

**Figure 6:**
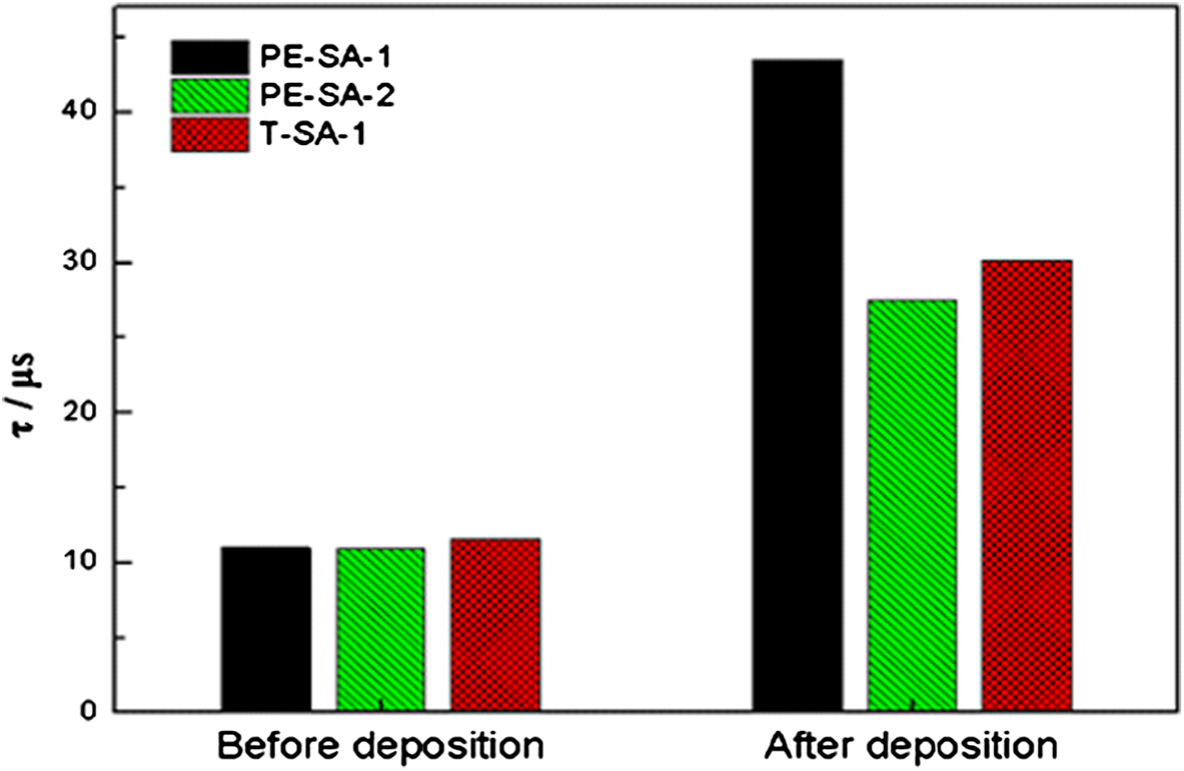
**Effective lifetime for single-side a-Si:H capped Si before and after Al**_**2**_ **O**_**3**_ **deposited.** The Al_2_O_3_ films were deposited on PE-SA-1 and PE-SA-2 using PE-ALD at 200°C. The Al_2_O_3_ films were deposited on T-SA-1 using T-ALD at 200°C. After Al_2_O_3_ film deposition, the samples were annealed at 450°C for 10 min in air.

Figure 6 shows that the effective minority carrier lifetimes of the a-Si:H(i)/Al_2_O_3_ stack samples increase by a factor of three (from about 10 μs to about 30 μs) compared with only a-Si film passivation samples. The increases in the effective minority carrier lifetime mean that the chemical passivation or field-effect passivation improved, which are dominated by Extended-Shockley-Read-Hall recombination at the Si surface. In this case after capping with Al_2_O_3_ films, the reduction of average *D*_it_ and the increase of negative charges weaken the trap effect of defects and reduce the interface recombination to improve the effective minority carrier lifetime. From the data above, a-Si:H(i)/Al_2_O_3_ stack also shows a good thermal stability at 450°C that can extend the a-Si:H(i) film application field in the solar cell fabrication.

## Conclusions

In this study, we demonstrated that the a-Si:H(i)/Al_2_O_3_ stack passivation layer can provide satisfactory passivation effect and thermal stability after annealing at 450°C for 10 min. The minimum interface defect density of samples with a-Si:H(i)/Al_2_O_3_ stack passivation after annealing was reduced from 3 × 10^12^ cm^−2^ · eV^−1^ (only with a-Si:H(i) film passivation) to 1 × 10^12^ cm^−2^ · eV^−1^; *Q*_tot_ increased by nearly one order of magnitude for PE-ALD samples and about 0.4 × 10^12^ cm^−2^ for T-ALD sample, and the effective minority carrier lifetimes increased by a factor of three (from about 10 μs to about 30 μs) compared with only a-Si:H(i) film passivation. Combining those passivation test results with the XPS analysis, we also discussed the oxide precursor influence of direct ALD Al_2_O_3_ films and a-Si:H(i)/Al_2_O_3_ stack on silicon passivation. The T-ALD Al_2_O_3_ films were low-density and hydrogen-rich films compared with PE-ALD Al_2_O_3_ films, leading to a low density of interface trap (excellent chemical passivation). The PE-ALD Al_2_O_3_ films also can provide excellent field effect passivation due to large number of negative charges. The results suggest the ALD Al_2_O_3_ film using H_2_O as O precursor could be helpful for hydrogen transport and interface hydrogenation, while the ALD Al_2_O_3_ film using O plasma precursor can be beneficial for negative charge formation. Furthermore, negative charge-rich Al_2_O_3_ films together with optimization of the a-Si:H(i) film deposition had more possibility to improve the a-Si:H(i)/Al_2_O_3_ stack passivation effect. The findings in this study can offer advantages for silicon passivation optimization of ALD Al_2_O_3_ films and the a-Si:H(i)/Al_2_O_3_ stack and improve the thermal stability of a-Si:H(i) films during solar cell fabrication process.

## References

[1] Taguchi M, Kawamoto K, Tsuge S, Baba T, Sakata H, Morizane M (2000). HIT™ cells—high-efficiency crystalline Si cells with novel structure. Prog Photovolt Res Appl.

[2] Dingemans G, Terlinden NM, Pierreux D, Profijt HB, van de Sanden MCM, Kessels WMM (2011). Influence of the oxidant on the chemical and field-effect passivation of Si by ALD Al_2_O_3_. Electrochem Solid-State Lett.

[3] Burrows MZ, Das UK, Opila RL, De Wolf S, Birkmire RW (2008). Role of hydrogen bonding environment in a-Si: H films for c-Si surface passivation. J Vac Sci Technol A.

[4] Dingemans G, Beyer W, van de Sanden MCM, Kessels WMM (2010). Hydrogen induced passivation of Si interfaces by Al_2_O_3_ films and SiO_2_/Al_2_O_3_ stacks. Appl Phys Lett.

[5] Dingemans G, Engelhart P, Seguin R, Einsele F, Hoex B, van de Sanden MCM (2009). Stability of Al_2_O_3_ and Al_2_O_3_/a-SiN*x*:H stacks for surface passivation of crystalline silicon. J Appl Phys.

[6] Seiffe J, Gahoi A, Hofmann M, Rentsch J, Preu R (2013). PECVD Al_2_O_3_/a-Si:B as a dopant source and surface passivation. Physica Status Solidi (a).

[7] Schutz-Kuchly T, Slaoui A (2013). Double layer a-Si:H/SiN:H deposited at low temperature for the passivation of N-type silicon. Appl Phy A.

[8] Dingemans G, Einsele F, Beyer W, van de Sanden MCM, Kessels WMM (2012). Influence of annealing and Al_2_O_3_ properties on the hydrogen-induced passivation of the Si/SiO_2_ interface. J Appl Phys.

[9] Veith B, Werner F, Zielke D, Brendel R, Schmidt J (2011). Comparison of the thermal stability of single Al_2_O_3_ layers and Al_2_O_3_/SiN*x* stacks for the surface passiviation of silicon. Energy Procedia.

[10] Veith B, Dullweber T, Siebert M, Kranz C, Werner F, Harder NP (2012). Comparison of ICP-AlO*x* and ALD-Al_2_O_3_ layers for the rear surface passivation of C-Si solar cells. Energy Procedia.

[11] Schmidt J, Merkle A, Brendel R, Hoex B, De Sanden MCM, Kessels WMM (2008). Surface passivation of high-efficiency silicon solar cells by atomic-layer-deposited Al_2_O_3_. Prog Photovolt Res Appl.

[12] Hofmann M, Schmidt C, Kohn N, Rentsch J, Glunz SW, Preu R (2008). Stack system of PECVD amorphous silicon and PECVD silicon oxide for silicon solar cell rear side passivation. Prog Photovolt Res Appl.

[13] Gatz S, Plagwitz H, Altermatt PP, Terheiden B, Brendel R (2008). Thermal stability of amorphous silicon/silicon nitride stacks for passivating crystalline silicon solar cells. Appl Phys Lett.

[14] Schaper M, Schmidt J, Plagwitz H, Brendel R (2005). 20.1%-efficient crystalline silicon solar cell with amorphous silicon rear-surface passivation. Prog Photovolt Res Appl.

[15] D’Amico J, Wilson M, Almeida C, Lagowski J, Olibet S (2013). Advanced interface trap metrology for silicon PV.

[16] Hoex B, Gielis JJH, van de Sanden MCM, Kessels WMM (2008). On the c-Si surface passivation mechanism by the negative-charge-dielectric Al_2_O_3_. J Appl Phys.

[17] van Hemmen JL, Heil SBS, Klootwijk JH, Roozeboom F, Hodson CJ, van de Sanden MCM (2007). Plasma and thermal ALD of Al_2_O_3_ in a commercial 200 mm ALD reactor. J Electrochem Soc.

[18] Liao HM, Sodhi RNS, Coyle TW (1993). Surface composition of AlN powders studied by x‐ray photoelectron spectroscopy and bremsstrahlung‐excited Auger electron spectroscopy. J Vac Sci Technol A.

[19] Zhu LQ, Liu YH, Zhang HL, Xiao H, Guo LQ (2014). Atomic layer deposited Al_2_O_3_ films for anti-reflectance and surface passivation applications. Appl Surf Sci.

[20] Xu Z, Zhu C, Huo Z, Zhao S, Liu M (2012). Effects of high-temperature O_2_ annealing on Al_2_O_3_ blocking layer and Al_2_O_3_/Si_3_N_4_ interface for MANOS structures. J Phys D Appl Phys.

[21] Li L-J, Zhu B, Ding S-J, Lu H-L, Sun Q-Q, Jiang A (2012). Three-dimensional AlZnO/Al_2_O_3_/AlZnO nanocapacitor arrays on Si substrate for energy storage. Nanoscale Res Lett.

[22] Savtchouk MW A, Edelman P, Lagowski J, Zhan X, Rong Y, Ted G (2014). Interface traps at intrinsic ia-Si:H/c-Si interfaces. 24th Workshop on Crystalline Silicon Solar Cells & Modules: Materials and Processes.

[23] Steingrube S, Steingrube DS, Brendel R, Altermatt PP (2010). Comprehensive model for interface recombination at a-Si:H/c-Si interfaces based on amphoteric defects. Phys Status Solidi C.

[24] Winer K (1990). Defect formation in a-Si: H. Phy Rev B.

[25] Powell MJ, Deane SC (1993). Improved defect-pool model for charged defects in amorphous silicon. Phys Rev B.

[26] Olibet S (2009). Properties of interfaces in amorphous/crystalline silicon heterojunctions.

